# Enhancing the implementation and sustainability of fundamental movement skill interventions in the UK and Ireland: lessons from collective intelligence engagement with stakeholders

**DOI:** 10.1186/s12966-021-01214-8

**Published:** 2021-11-03

**Authors:** Jiani Ma, Michael J. Hogan, Emma L. J. Eyre, Natalie Lander, Lisa M. Barnett, Michael J. Duncan

**Affiliations:** 1 Centre for Sport, Exercise and Life Sciences, Faculty of Health and Life Sciences, Coventry University, Coventry, UK; 2 School of Health and Social Development, Institute for Physical Activity and Nutrition, Deakin University, Melbourne, Australia; 3 School of Psychology, National University of Ireland, Galway, Ireland; 4 Institute for Physical Activity and Nutrition, School of Exercise and Nutrition Sciences, Deakin University, Melbourne, Australia

**Keywords:** Physical activity, Motor competence, Motor skills, Implementation science, Systems science, Child, Adolescent, Physical education

## Abstract

**Background:**

To have population-level impact, physical activity (PA) interventions must be effectively implemented and sustained under real-world conditions. Adequate Fundamental Movement Skills (FMS) is integral to children being able to actively participate in play, games, and sports. Yet, few FMS interventions have been implemented at scale, nor sustained in routine practice, and thus it is important to understand the influences on sustained implementation. The study’s aim was to use Collective Intelligence (CI)—an applied systems science approach—with stakeholder groups to understand barriers to the implementation of FMS interventions, interdependencies between these barriers, and options to overcome the system of barriers identified.

**Methods:**

Three CI sessions were conducted with three separate groups of experienced FMS intervention researchers/practitioners (*N* = 22) in the United Kingdom and Ireland. Participants generated and ranked barriers they perceive most critical in implementing FMS interventions. Each group developed a structural model describing how highly ranked barriers are interrelated in a system. Participants then conducted action mapping to solve the problem based on the logical relations between barriers reflected in the model.

**Results:**

The top ranked barriers (of 76) are those related to policy, physical education curriculum, and stakeholders’ knowledge and appreciation. As reflected in the structural model, these barriers have influences over stakeholders’ efficacy in delivering and evaluating interventions. According to this logical structure, 38 solutions were created as a roadmap to inform policy, practice, and research. Collectively, solutions suggest that efforts in implementation and sustainability need to be coordinated (i.e., building interrelationship with multiple stakeholders), and a policy or local infrastructure that supports these efforts is needed.

**Conclusions:**

The current study is the first to describe the complexity of barriers to implementing and sustaining FMS interventions and provide a roadmap of actions that help navigate through the complexity. By directing attention to the ecological context of FMS intervention research and participation, the study provides researchers, policy makers, and practitioners with a framework of critical components and players that need to be considered when designing and operationalising future projects in more systemic and relational terms.

**Supplementary Information:**

The online version contains supplementary material available at 10.1186/s12966-021-01214-8.

## Background

Increased physical activity has been associated with health benefits in young people (aged 5–17 years), including improved physical, psychosocial and cognitive outcomes [1]. Nonetheless, young people worldwide are not engaging in sufficient levels of physical activity as recommended [1]. Physical activity is a multidimensional movement behaviour [2]. For young people, it consists of play, games, sports, transportation, chores, recreation, physical education (PE) or planned exercise [2]. Underlying successful participation in these activities is competence in basic movement patterns such as running and catching–known as Fundamental Movement Skills (FMS) [3]. Enhancing children’s FMS proficiency contributes to maintaining a physically active lifestyle [4, 5]. Therefore, the World Health Organization recommended promoting FMS development in children in recent physical activity guidelines [6]. Yet, young people’s FMS levels are low worldwide—a recent systematic review that synthesised FMS data from 25 countries revealed that children are not achieving FMS competence required to successfully participate in physical activity [7]. This has occurred in spite of the preponderance of interventions reported as improving FMS (see [8–11] for reviews). This suggests these interventions have rarely been implemented into routine practice to achieve sustained population-level impacts [12].

Translating effective research to practice requires understanding of implementation and sustainability [13]. Implementation of an intervention refers to the process of integrating an intervention into practice within organisations and settings [14]. The extent to which the programme can continue to be delivered over time and institutionalised within settings is defined as sustainability [15]. Ineffective/inadequate implementation and sustainability in FMS interventions need to be addressed but have received little attention [16]. Whilst a handful of follow-up of FMS interventions have been performed [8, 17, 18], there has been little investigation of the factors that influenced implementation and sustainability of such interventions [16]. Lander et al. [19] found a variety of barriers and facilitators that influenced the sustained implementation of a school-based programme three years post intervention. Higher levels of teacher’s efficacy to teach and assess FMS, curriculum alignment, and student’s engagement were highlighted as facilitators that support ongoing implementation [19]. The breadth of the study findings provide valuable insights into the potential mechanisms to expand FMS programme impact, but did not account for the complex interdependencies of the influences on the implementation and sustainability of FMS interventions. The need to understand the system of interdependent influences is grounded in the nature of FMS development—a complex and dynamic process, that is characterised by the interaction of a child’s maturation with external factors such as physical and social contexts, that support continuous and adaptive experience of movement [20]. This developmental complexity highlights the need for further examination on the underlying mechanisms and contextual constraints of sustained FMS intervention implementation success. This examination needs to be situated in a broader ecological system within which a multitude of influences operate across individual, organisational, community, and systems levels [13, 21]. These influences are interrelated and dynamic in nature [22]. For instance, despite the recognition of the importance of FMS development in PE curricula worldwide [23], the marginal status of PE compared to other core subjects limits the opportunities for children to develop skills needed [24]. The operation of these and other contextual constraints highlights the need for systems-based investigations of FMS interventions that account for the contextual complexity within which FMS development occurs [25].

Systems thinking is an emerging approach to understand intervention scenarios and the dynamics. It has recently been recommended as a means to enhancing intervention implementation and sustainability [26] by examining the interconnectedness of key components in an intervention [27]. The application of systems thinking in health interventions is emerging, although this thinking has received critique for its limited reflection on what it might mean for the development and evaluation of interventions [26].

One applied systems science approach–Collective Intelligence (CI)–has been widely used to facilitate group-based problem solving, specifically, to both understand a complex issue and map options and actions relevant to the problem [28] (see [29–32] for recent social science applications; and see [33–35] for further details on methodology and application). The benefits of applying this approach to understand the complexity of FMS interventions has recently been outlined in an exploratory study [36]. For example, CI helped the stakeholder group to map and understand the relationship between barriers to the implementation of FMS interventions. A major strength of the CI method used in the current study is the way in which key systems thinking products can be combined from across multiple group design sessions. Application of the CI method results in the production of a matrix-based structural map (i.e., a systems thinking output) generated from collective, deliberative input from a group, which allows for a meta-analytical examination of multiple structural maps that combine the ideas and reasoning across multiple sessions. This allows for a synthesis of perspectives and the development of an integrated roadmap that can be used to inform practical recommendations and enhance sustainability of strategies to promote FMS in children and adolescents in various contexts [23].

Considering the need to translate effective research into practice to enhance FMS proficiency at the population level, and given the complex nature of FMS interventions, the current study sought to understand barriers to the implementation and sustainability of FMS interventions, interdependencies between these barriers, and options to address the system of barriers identified. To do so, we used CI with three stakeholder groups in the UK and Ireland who have expertise in FMS interventions. To our knowledge, this will be the first meta-analytical examination of FMS implementation issues and the first time an applied systems science approach is used to identify barriers and their interdependencies along with options to address these barriers.

## Methods

### Participants

A purposeful sampling adopting a ‘criterion-I’ strategy was used [37]. This strategy is commonly applied in studies that seek to engage participants from organisations and systems involved in the implementation process. The criterion used in our study are also consistent with the prerequisites for the optimal outcome of CI sessions [38], in particular, engaging with stakeholders and content specialists who have a stake in the issues being considered (i.e. school teachers, coaches, FMS researcher, public health specialists). This was done by identifying individuals named in publications/reports associated with FMS interventions in the UK and Ireland. A snowball sampling technique was also used to identify additional individuals that had a significant role in the intervention setting. Twenty-two participants were conveniently recruited across Location A^1^ and B in the UK and Location C, Ireland (Table 1). The selection assumed that the individuals and the organisations they are embedded in possess expert knowledge of FMS intervention implementation by virtue of their experience in developing, implementing, delivering and evaluating FMS interventions. Most individuals worked across both academic institutions and local intervention practice settings, and thus were in a position to provide information that is both detailed and generalisable across the lifecycle of FMS intervention project work. Individuals were contacted via e-mail/telephone and provided with a plain language statement. All participants provided their written consents prior to engaging in the CI process. Ethics clearance was granted by Ethics Committees of Coventry University (P90462) and Deakin University (HEAG-H 173_2020).

**Table 1:** Key stakeholder characteristics

	Session 1 (UK, *N* = 5)	Session 2 (Ireland, N = 6)	Session 3 (UK, N = 7)
Number of males/females	2/3	3/3	1/6
Primary profession: - Academic	5	1	7
- Schoolteacher	0	3	0
- Health promotion officer	0	2	0
Subject areas participants have expertise/experience in %(N)			
Physical Education	40(2)	83(5)	43(3)
Sports Coaching	60(3)	17(1)	29(2)
FMS intervention design and evaluation	80(4)	67(4)	100(7)
FMS intervention delivery	80(4)	83(5)	100(7)
Public health specialists	40(2)	33(2)	29(2)
Primary/Secondary school teaching	20(1)	50(3)	29(2)

### Data collection

**Fig. 1:**
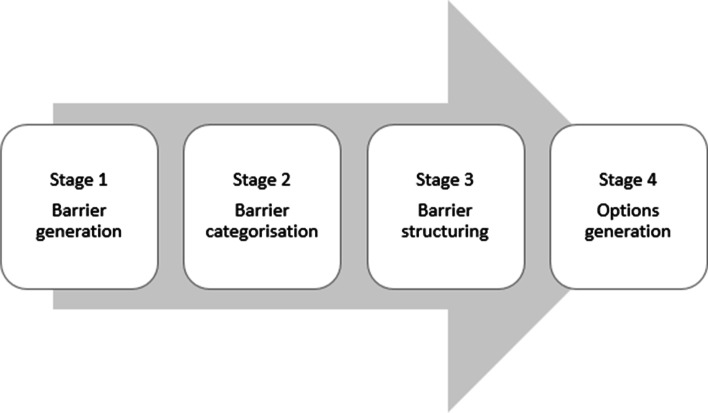
The four-stage Collective Intelligence process

CI is a facilitated group consultation process designed for collective problem-solving [28, 29, 33, 39]. Given the novelty of this approach in the context of FMS research, a protocol has been recently published to detail its rationale, procedures, and benefits [36]. In summary, the same four-stage process (Fig. 1) as described in the protocol paper was used in the current study. Stage one involved inviting all 22 participants to generate five barrier statements in response to a trigger question delivered via email: “*From your understanding and previous involvement in FMS interventions, what do you consider are the key barriers to the adoption, implementation and institutionalisation of effective FMS interventions?*” These barriers were collated and categorised by the CI facilitation team in Stage two. Stage three involved a closed voting process in which each participant was asked to select seven barriers they perceived as most critical across the categorised barrier field. Barriers which received most votes were considered most critical and these barriers were then structured using *Interpretive Structural Modelling* (ISM [28];) software in a facilitated process with participant groups during pre-organised CI sessions. This structuring process was detailed in [36] and involves a process of matrix structuring. In particular, to generate a systems model derived from a matrix of relations between barriers, the relationship between pairs of barriers was explored in a facilitated dialogue focussed on the reasoning of participants, prompted in each case using a relational question, “*Does Barrier A significantly aggravate Barrier B?*”. After reasons and objections were considered by participants, a vote was taken to determine the group’s judgment about the relationship (i.e., either ‘yes’ or ‘no’ to the relational question). Upon completion of all pairwise judgements, the matrix of relations is converted by ISM software into a graphical systems model illustrating relations across the set of barriers. In Stage four, during the CI sessions, participants engaged in a process of generating options for overcoming the barriers identified. In this stage, participants were asked to generate, clarify, and present options according to the ISM structural map, in particular, focusing on key driver barriers and associated categories that were identified to significantly aggravate other barriers in the system. Overall, the CI sessions each lasted approximately five hours.

Three sessions were conducted between December 2019 and November 2020 with three separate FMS project teams in the UK and Ireland. These sessions were facilitated by the lead author and/or Author 2. Each session was scheduled at a time that was convenient to the majority in the participant group and some participants were not present due to unavailability (4/22, 18.2%). CI sessions one (*N* = 5) and two (*N* = 6) were conducted face-to-face on a university campus accessible to all participants, and session three (*N* = 7) was conducted online via Zoom due to pandemic restrictions. The lead author took field notes during and following each session. Field notes are an essential component of rigorous qualitative research and used to capture contextual information of the data collection and aid understanding of the outcome [40]. Consideration was given to observations of paired comparison that required extended discussions and reflective data including researcher thoughts and ideas relating to the group discussion and reasoning.

### Data analysis

The analysis and reporting process used in the current study follows the standard processes of generating categorised field representations of ideas and for meta-analysis of ISM structure as described in [41]. Barrier statements in response to the initial trigger question collated from participants were analysed by barrier categories. For each of the three CI sessions, participants voted to select barriers for structuring from across the category field and were facilitated to generate a structural model through a process of deliberation and matrix structuring in the session. Each structural map was analysed in conjunction with field notes. Additionally, a structural meta-analysis of the three models was conducted to understand the relationship between categories of barriers and to identify high-level structural relations emergent across the three FMS intervention scenarios. The meta-analysis process is therefore described in the results section, following presentation of three structural models.

Option statements generated in response to barriers categories were collated and analysed. These option statements were then summarised by Author 1 and Author 2 to generate synthesised options. These options were then thematically categorised based on conceptual clusters aligned with the Expert Recommendations for Implementing Change (ERIC) framework [42, 43]. The ERIC framework provides a compilation of strategies to improve implementation of interventions and has been used to advance school-based intervention research [44]. Mapping options generated in this study with the ERIC framework enables a systematic recommendation on particulars that can be taken to improve implementation and sustainability of FMS interventions. This also follows good practice in implementation science by advocating the consistent use of theoretical frameworks and terminology [45].

## Results

### Category analysis of barriers

The three sessions generated a total of 76 barriers. These barriers were categorised using the paired-comparison method ([28]; cf. [41]). The CI facilitation team (Author 1 and Author 2) conducted open coding and category creation. Specifically, pairs of barriers were systematically assessed for conceptual similarity and conceptually similar barriers were grouped under higher-order categories. This process is continued until all ideas have been placed into final categories. The facilitation team followed this process and identified 13 categories (see Table 2). Table 2 also provides a description of each category of barriers, along with sample ideas in the category.

**Table 2:** All 13 categories of barriers generated from the CI process

Category	Clarification	Sample statements from CI process
A. Time	Time constraints to integrate the proposed programme	A demand for time in the curriculum, impacting time allotted for interventions
B. Government and Institutional	Factors relating to policy that may support institutionalisation of the programme.	Refusal of government to offer greater time for PE/sports in schools
C. Curricular Conflicts	The contextual appropriateness and congruence with the existing curriculum and schools’ priorities	Conflict between school targets and research targets
D. Design and Implementation	The compatibility and adaptability of the proposed programme	Lack of considerations of long-term sustainability and implementation of the programme
E. Research Challenges	Challenges relating to conducting intervention research	Failure to recruit schools/children to interventions
F. Knowledge and Appreciation	Perceived need and benefits of the proposed programme and possession of the relevant skills and knowledge	Lack of teacher knowledge of FMS and PA in children
G. Conflicts and Purposes within PE	The contextual appropriateness and congruence with the current PE curriculum and practice	Conflicting interpretations among PE teachers of the aims and the purpose of FMS interventions
H. Resources and Funding	Factors relating to funding and resources at the government level and individual organisational level.	Lack of funding to support implementation phase
I. Staffing	Specific considerations on staffing, internal advocates and managerial support necessary for successful implementation	Shortage of staff to support interventions, therefore prevents the ‘adoption’ of an intervention going forward
J. Efficacy and Attitude	Motivation and self-efficacy to implement the proposed programme	Unwillingness by PE teachers to implement strategies that they are not familiar with
K. Training	Approaches to insure providers proficiencies in the skills and knowledge required to implement the programme	Lack of Continuing Professional Development for PE teachers (i.e. minimal contact time with PE teachers) and therefore inadequate training
L. Testing Challenges	Challenges relating to conducting outcome assessments	Failure of test subjects to engage with demonstration from researchers
M. Intervention Evaluation	Practice and knowledge on programme evaluations.	Inadequate reporting on interventions, such as intervention process, actual ‘on-task’ time for FMS practice, and actual delivered dose of the intervention

**Fig. 2:**
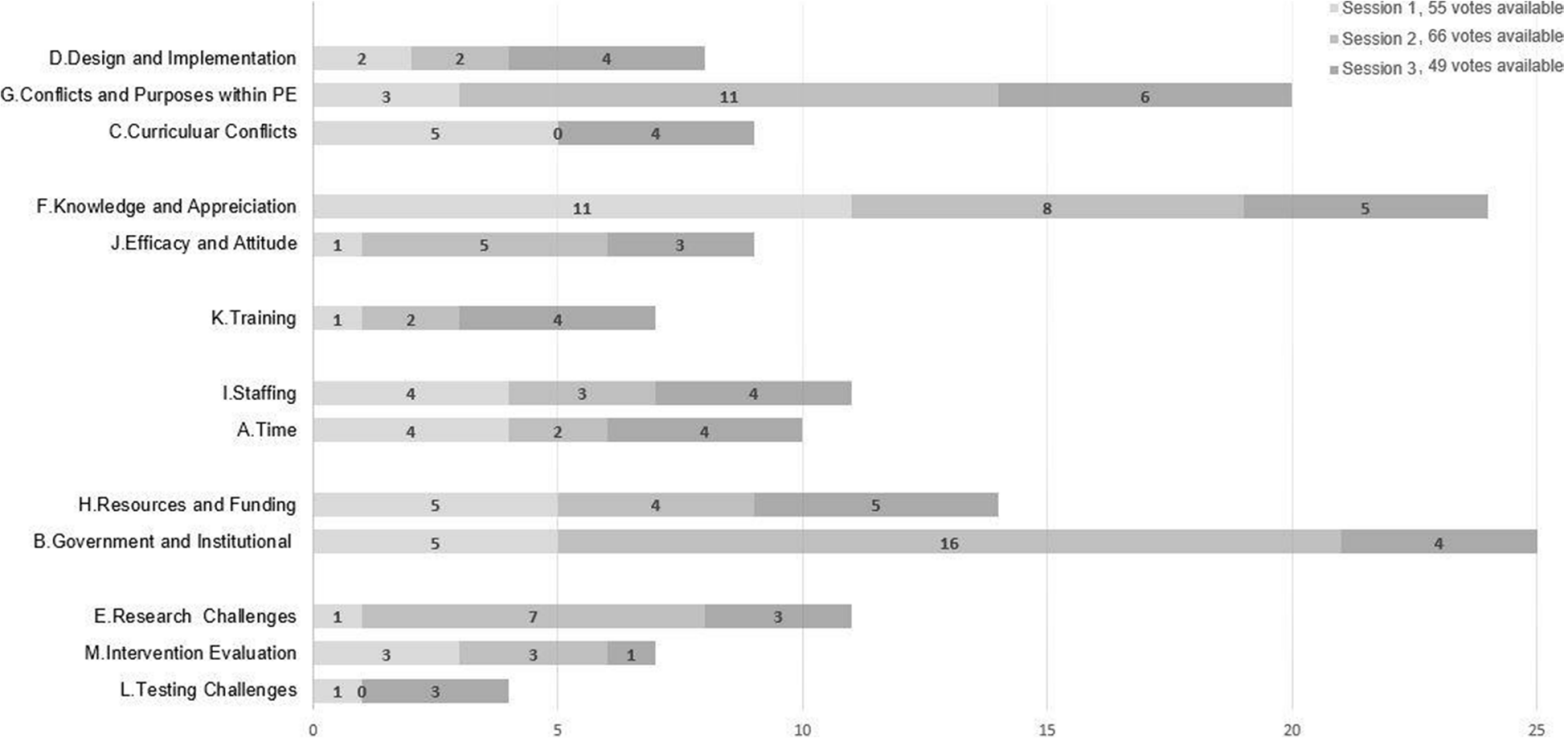
Votes received from each session, by barrier category

When selecting barriers for inclusion in the ISM structuring, participants each voted independently for seven critical barriers, with a total number of aggregate votes at the group level reflecting the perceived importance of barriers. The total number of votes per category received from each session is presented in Fig. 2. Category [F. Knowledge and Appreciation], [B. Government and Institutional] and [G. Conflicts and Purposes within PE] received most votes from Group 1, Group 2, and Group 3, respectively. These three categories also received most cumulative votes from the three groups collectively. Group 2 did not identify any critical barriers in Category [C. Curricular Conflicts] and [L. Testing]. Category [L. Testing] received the least votes across three groups.

### Structural models generated in each session

A brief description of structural models generated from each session is presented below. Structures are to be read from left to right, with the barriers on the left significantly aggravating (i.e., make worse) the barriers to the right. Barriers grouped together in one box are reciprocally interrelated and they significantly aggravate each other.

#### Session 1

**Fig. 3:**
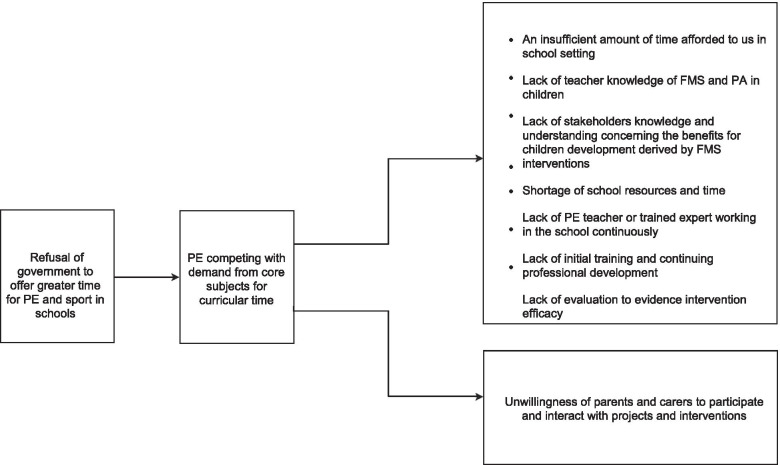
Barrier structure from Session 1. Structure are to be read from left to right, with the barriers on the left significantly aggravating (make worse) the barriers to the right. Barriers are grouped together in one box are reciprocally interrelated and they significantly aggravate each other

Five participants attended Session 1 and generated a model of 10 barriers (Fig. 3). In the model, *“Refusal of government to offer greater time for PE and sport in schools”* was considered to be a fundamental driver of all other barriers. It was argued that this barrier further aggravates *“PE competing with demand from core subjects for curricular time”*, which further influenced all other barriers, including barriers to engaging parents and carers, and seven reciprocally interrelated barriers (i.e., time and resources constraints, insufficient knowledge of teachers and stakeholders, lack of training, lack of continued implementation, and lack of evaluation evidence).

During the pair-wised reasoning, one main emergent theme was challenges associated with practice on school grounds, including teachers’ knowledge, time and resources to support delivery. Notably, participants recognised the cyclical relationship among these factors and also judged these barriers are the result of *“PE not being recognised as a core subject”*. Interestingly, participants also agreed this influences parents and carers willingness to interact with interventions. Participants reasoned that, without continued practice outside the school environment, children would not have sustained improvement of skills from the intervention. Barriers related to Efficacy and Attitude were not perceived as critical, particularly in comparison to barriers related to Knowledge and Appreciation. Participants argued deliverers’ (e.g., teachers) attitudes towards the intervention are largely driven by their understanding and subject knowledge of FMS which influence perceived benefits of the intervention.

#### Session 2

**Fig. 4:**
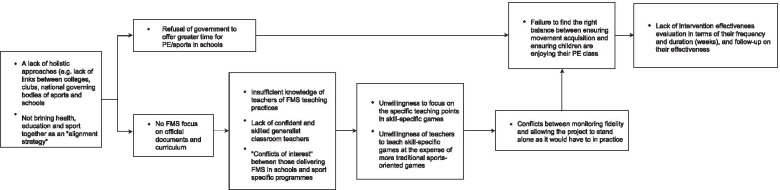
Barrier structure from Session 2. Structure are to be read from left to right, with the barriers on the left significantly aggravating (make worse) the barriers to the right. Barriers are grouped together in one box are reciprocally interrelated and they significantly aggravate each other

Six participants attended Session 2 and generated a model of 12 barriers (Fig. 4). This group considered the lack of school/community holistic approaches and the misalignment between health, education, and sports as interrelated and critical drivers of the barrier system. Aggravated by these critical drivers are the lack of government supports and FMS curriculum focus. These barriers spill over into teacher’s understanding and appreciation of FMS, which, in turn, impact teaching practice and intervention effectiveness. Participants also attributed teacher’s unwillingness to focus on FMS to teacher’s insufficient FMS content knowledge and pedagogical content knowledge, as well as poor self-efficacy in this area. These two sets of barriers were considered to be caused by the lack of focus on FMS in official documents and curriculum.

#### Session 3

**Fig. 5:**
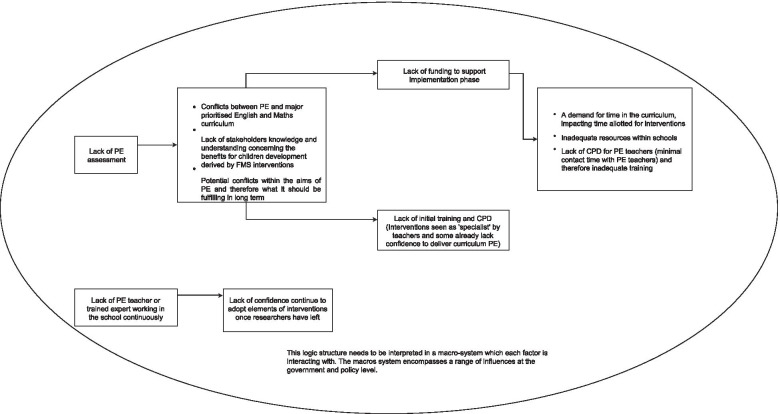
Barrier structure from Session 3. Structure are to be read from left to right, with the barriers on the left significantly aggravating (make worse) the barriers to the right. Barriers are grouped together in one box are reciprocally interrelated and they significantly aggravate each other

Seven participants attended Session 3 and generated a model of 11 barriers (Fig. 5). This group identified two barriers as fundamental drivers of negative influence in the system. The first was the *“Lack of PE assessment”*. The group agreed that the absence of PE assessment is central to curriculum conflicts and impacts negatively on stakeholder and teacher’s perceived benefits of FMS interventions. This barrier further led to the lack of funding and training opportunities to support intervention implementation. The lack of funding was considered to aggravate the time pressure in delivering interventions and providing ongoing support to teachers, as well as to limit availability of resources within schools. Another fundamental driver was “Lack of PE teacher or trained expert working in the school continuously”, which resulted in teacher’s lack of confidence to continue intervention delivery.

Notably, when participants reviewed the ISM structure, debates emerged as regards other overarching influences. Participants argued that their structure needs to be interpreted “*in the context of a wider system*”, specifically, in relation government and policy influences. The group arrived at a consensus that barriers in their structure *“needs to be addressed at the macro level before a meaningful long-term change can be made at the micro level”.*

### Meta-analysis of three structural models: influence map of barriers

Barriers from across 10 of the 13 categories appeared in the three ISM structures. A structural meta-analysis of the three models was conducted to understand the relationship between categories of barriers. In order to carry out this analysis, the following scores (i.e., position score, antecedent/succedent score, influence score) were computed to estimate the influence of each category, as per reported process in [41].

#### Position score

Each structural map places barriers in levels (i.e. the columns barriers are positioned in) [46]. Ideas to the far right are assigned the lowest position score (i.e., 1), and those in the leftmost position are assigned the highest score (i.e., depending on the number of levels in the structure). For instance, in the structural map generated in Session 1 (Fig. 3), there are three levels; the idea to the far left is assigned a score of 3, ideas to the far right are assigned a score of 1.

#### Antecedent and succedent score

The antecedent score is the number of barriers lying to the left of a particular barrier that aggravates it. The succedent score is the number of barriers lying to the right of a barrier in the structure that are aggravated by it. The net succedent/antecedent (net SA) score is the succedent score minus the antecedent score. A positive net SA score indicates the barrier is a net aggravation influence. A negative net SA score indicates the barrier is a net receiver of aggravation [46].

#### Influence score

**Fig. 6:**
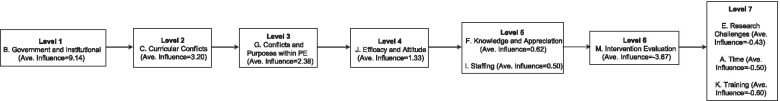
Meta-analysis of three influence structures (meta-structure)

The influence score is the sum of the position score and the net SA score. Influence scores were calculated for each of the 33 barriers appearing in the three structural models. Total category influence scores were calculated by summing the individual barrier scores. Average category influence scores were calculated by dividing this total category influence score by the number of barriers in the category. The meta-analytical model arranges barrier categories from left to right based on their average influence scores (see Fig. 6).

### Analysis of options

Based on relationship between barriers in their ISM structure, each CI group generated options to overcome barriers in relevant barrier categories. Participants used the idea writing technique [28] to generate and share ideas. Participants generated option statements on a set of shared sheets, with opportunity to add ideas as they silently read the ideas written by others. Participants generated option statements starting with action verbs, such as *create, develop, encourage, plan*.

**Fig. 7:**
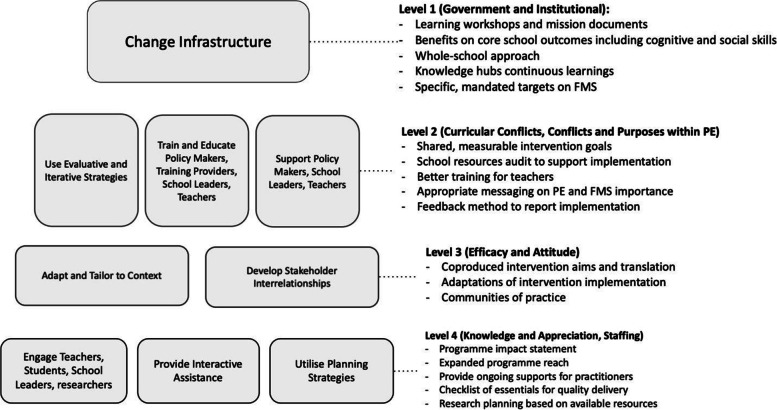
A roadmap of actions to overcome barriers in implementing and sustaining FMS interventions

In total, 125 option statements were generated across three sessions. Options were generated targeting barriers across Level 1 to Level 5 in the metal-analytical influence map (Fig. 6). Barriers across Level 1 to Level 5 have positive influence scores, which indicates that they are net aggravation influences in the barrier system that need to be prioritised. All option statements and the associated barrier category they address are presented in Additional file 1. During analysis, these option statements were clustered into 38 higher level synthesised solutions, which were further categorised into nine conceptual clusters linked to the ERIC (see Table 3). To adapt to the context of FMS intervention research for future dissemination, we made surface changes to terminology, which are noted in Table 3. Focusing on Level 1 to Level 5 in the meta-analytical influence map (Fig. 6), each solution was assigned a score corresponding to the level of the barrier it aims to address in the influence map that represented the solution’s potential to address barriers across the field. For instance, solutions designed to overcome barriers relating to [B. Government and Institutional] (i.e., Level 1 in Fig. 6, which includes barriers with the highest net aggravating influence) were assigned a score of 5. This high score of 5 corresponds to the high level of potential influence of solutions addressing Level 1 barriers. Following this scoring method and logic, a solution addressing barriers in Level 2 was assigned the next highest score of 4, a solution addressing barriers in level 3 was assigned a score of 3, and so on. After all solutions were scored, and a roadmap representing a hierarchy of actions that corresponds to the barrier meta-structure was developed (Fig. 7). The roadmap reads from top to bottom with a synthesis of essential activities described on the right in each level. Level 1 actions target barriers at the government and institutional level (Category B) and include activities to create and improve infrastructures. These actions are considered most influential in resolving barriers to implementing and sustaining FMS interventions. Level 2 actions correspond to the barriers associated with curricular conflicts (Category C) and purposes within PE (Category G). Actions focus on training and supports provided to multiple change agents, as well as strategies that researchers and practitioners can employ to monitor and evaluate programme implementation. Level 3 actions are designed to overcome various disincentives to engage in interventions (Category J), with emphases on implementation adaptations and stakeholder interrelationships. At Level 4, there are three sets of solutions to enhance intervention user’s knowledge and appreciation (Category F) and to alleviate negative influences from practical challenges relating to staffing (Category I).

**Table 3:** Strategies to implementing and sustaining FMS interventions

Original Label for the Strategy Cluster	Adapted Label Strategy Cluster	Implementation strategies falling under each strategy cluster
Engage Consumers*	**Engage teachers, students, school leaders, researchers**	Report impact from the programme and disseminate knowledge in relation to quality of life, health, and learning outcomes^c^
		Promote publicity and impact of the intervention programme to potential stakeholders and build reciprocal relationships with them to involve them in future research^e^
		Expand programme reach to parents and mobilise parental engagement in interventions^f^
Use Evaluative and Iterative Strategies	No change	In advance of programme implementation, generate shared, measurable goals in a collaboration between schools, researchers and policy makers, and build coalitions and partner relationships to support implementation efforts^c^
		Evaluate, adapt, and create the physical structures, equipment, and school resources to support programme implementation^c^
		Improve and change the current evaluation practice to incorporate more appropriate techniques, change the priority of what determines an intervention success and conduct more long term and follow-up evaluation to monitor sustainability^b^
		Conduct more rigorous and comprehensive evaluation including pilot research, long term follow-up that yields sustainability data, and evaluation of what determines intervention success^m^
		Conduct research on participant understanding of and engagement in intervention programmes and create solutions to overcome perceived barriers and misconceptions^m^
Change Infrastructure	No change	Change school ethos and values around PE through learning workshops and mission documents that promote awareness and understanding of FMS and its impact on core school outcomes including cognitive and social skills^c^
		Use and promote a whole-school approach to embed movement opportunities throughout the whole school day, including curricular, extracurricular, cross-curricular, active transport, and homework^c^
		Establish a multi-sector task force to develop, implement, and evaluate child health and development policies and programmes that support PE in schools by directing appropriate funding and resources to local councils^b^
		Develop structures to support programme sustainability, including developing knowledge hub and partner relationships, educating undergraduates, and promoting programme integration into curriculum^b^
		Establish specific, mandated targets on FMS and PA and demand these to be achieved and reported by schools, in order to direct intervention time and resources and encourage programme uptake by schools^c^
		Challenge the idea of correct technique in children’s movement and encourage children to explore under guidance^f^
		Encourage integration of programmes and interventions with pre-existing school curriculum and syllables^m^
		Integrate intervention science and associated field work in undergraduate teaching programmes^e^
		Create norms of knowledge building and continuous learning to support students, teachers, parents, and coaches^f^
Adapt and Tailor to Context	No change	Develop theory-based interventions and resources as well as adapt pedagogical approaches^b^
		Apply and prioritise PE/skills assessment for children and provide context-specific feedback to allow them to reflect on their progress and performance^g^
Develop Stakeholder Interrelationships		Build collaborations between research, schools and policy holders to promote joined-up thinking^b^
	No change	Conduct stakeholder to clarify intervention aims and results and consult stakeholders on ways to translate intervention findings into practical settings^g^
		Establish cross-disciplinary collaborations in research to access new tools, methods and expertise^e^
		Promote collaborations between research institutes for wider impact^m^
		Create communities of practice among research institutes and consult stakeholders on bids for funding^h^
Utilize Financial Strategies	**Utilise planning strategies**	Create a checklist of essentials for quality PE which guides schools planning on provisions^h^
		Conduct research planning based on available resources including proposing suitable research questions, creating cost-effective solutions in research activities such as training teachers to collect research data^h^
Support Clinicians	**Support policy makers, school leaders, teachers**	Build and communicate robust evidence with stakeholders to encourage uptake of PE and FMS at government level^b^
		Establish a feedback method for teachers to report fidelity on programme delivery^e^
		Promote common outcome metrics in PA and FMS across all stakehodlers^m^
		Translate evidence base into practical solutions coupled with evaluation techniques and measurable outcomes to create clear FMS guidelines, programme methods, and assessments to be embedded in PE curriculum^b^
		Create practical and appropriate resources and build structures to promote continuity of FMS messages following a life span approach and provide practitioners confidence and rewards to carry out ideas^g^
Provide Interactive Assistance	No change	Provide support for practitioners and teachers to co-lead the delivery of projects^g^
		Create a learning collaborative for stakeholders to share their knowledge and experience regarding FMS and existing FMS resources, as well as to link with researchers to disseminate importance of FMS and best practice^f^
Train and Educate Stakeholders	**Train and Educate policy makers, training providers, school leaders, teachers**	Promote recognition and importance of PE and FMS at national and local level through educating policy holders based on evidence drawn from high quality research^b^
		Demand and organise better training for teachers^b^
		Strengthen CPD for teachers and include intervention and educational aims in the training^g^
		Create appropriate resources and disseminate them in different formats to be shared with stakeholders, including guidelines on creating suitable skill learning environments, fun games for children to practice FMS, social marketing of programme benefits on children’s development and skill specific curriculum programmes^g^
		Plan and implement effective pre-service and in-service teacher training programme to include relevant pedagogies and techniques, learning workshops on knowledge and understanding of FMS^f^

*The nine higher-level themes of strategies are based on the conceptual categories of the Expert Recommendations for Implementing Change (ERIC) (Waltz et al., 2015). Superscripts stand for which barrier categories the solution is generated in response to. b, Government and Institutional; c, Curricular Conflicts; e, Research Challenges; f, Knowledge and Appreciation; g, Conflicts and Purposes within PE; h, Resources and Funding; m, Intervention Evaluation

## Discussion

This is the first study to use CI methodology to identify, rank, categorise, and structure relations between barriers related to implementation and sustainability of FMS interventions, and offer solutions. The study has provided an understanding of needs, expectations, and factors relevant to the implementation and sustainability of FMS interventions. Participants identified 76 barriers which were structured and analysed to provide an influence map of barriers and their inter-relationships. The top ranked barrier categories were: Category [B. Government and Institutional], [G. Conflicts and Purposes within PE] and [F. Knowledge and Appreciation]. Analysis of the structural models further revealed other influential barrier categories [C. Curricular Conflicts] and [J. Efficacy and Attitude]. Together, these five barrier categories consistently emerged as influential sources of aggravating influence across all three groups included in the current study and are present in the first five levels in the meta-structure (Fig. 6). While a number of studies have focused on factors that impede intervention success, less often have solutions been proposed to address a system of barriers in an applied context [26]. Our study provides solutions designed to address barriers and presents these solutions in the form of a roadmap that corresponds to the system of barriers and associated interdependencies (Fig. 7). These results are discussed below by reference to their relevance for policy, research, and practice in the implementation and sustainability of FMS interventions.

### Implications for policy

Our barrier analysis confirms that barriers originated from multiple levels and agents that are important to consider when implementing and sustaining FMS interventions in practical settings. Notably, barriers associated with government and institutional policy (Category B) can influence curricular related barriers (Category C) which further aggravates barriers related to individual knowledge and attitude (Category J and F) (as shown in Fig. 6). This is consistent with a previously proposed ecological model of influences on intervention implementation which reported that community/systems level factors (Category B) have overarching influences on practice at an organisational (Category G) and individual level (Category F) [13]. Our findings suggest that the lack of specific and measurable targets for PE and FMS in schools makes it challenging to divert the focus from core subjects such as Maths and English. This is a direct consequence of the educational focus of schools which is stipulated by the national education standards. Therefore, mandated changes need to be created for specific school targets on FMS and physical activity accompanied by a surveillance and report system, as well as alignment of PE curriculum and assessment. Setting quantifiable and comparable targets is essential to successful health policies [47]. In the current study context, evaluations on performance related to FMS learning and teaching and the accountability system for these to be achieved and reported on may direct the change in school’s ethos and values around PE. Via this mechanism (i.e., *Change Infrastructure*, see Fig. 7), effective FMS development strategies are more likely to be embedded into the school educational practice.

Government and national strategies need to facilitate this by advocating for a quality assurance system and by providing guidance to ensure PE is accorded the same status as other subjects. The pathway to this policy change presents strategic challenges. As an example, UK policy initiatives produced top-down funding streams (e.g., Pupil Premium for PE) to support this mission. There are official bodies (e.g., Ofsted in the UK) that develop metrics that can hold schools accountable for educational standards, which includes providing judgements on the overall effectiveness on the use of the funding to support school PE [48]. However, funding were used to employ external sports coaches to deliver PE rather than strategically developing school capacity to deliver quality PE [49]. Consistent with this, our findings suggest despite the best intentions, the local implementation of policy varies. The United Nations Educational, Scientific and Cultural Organization’s (UNESCO) recent initiative to promote Quality Physical Education worldwide, reported that policy changes are most effective when accompanied by cohesive and tangible demands [50]. Our roadmap proposes several specifics on setting the agenda to shape the policy process and policy content on PE. Noteworthy is participants’ recognition of autonomy at local levels. In the case of FMS interventions, creating and showcasing best practice and benefits for teachers, school leaders, and other stakeholders is recommended. Local change agents also need to be mobilised to create joint efforts, including parents, community sports clubs, governing bodies of sports, public health and education specialists, and research institutions. This wider group of stakeholders plays a key role in creating and maintaining social and physical environments that are conducive to children’s FMS development [51].

### Implication for practice

Our findings suggest, in practice, sustainable changes are likely to occur when interventions change the whole-school ethos and values that support intervention missions and PE provisions (Level 1 actions in Fig. 7). Therefore, central to this set of options is to promote a whole-school approach that embeds movement opportunities in children’s school as well as out of school time (i.e., PE, curricular lessons, extracurricular activities, active travel, and homework). This is consistent with the Creating Active School Framework which advocates to establish whole-school practice and ethos that informs beliefs, customs, and practices [24]. Specific to FMS development, a whole-school approach is a logical step to creating movement culture that comprises multiple forms and purposes [52].

School leadership (e.g. principals and head teachers) influences the quantity and quality of movement opportunities [53, 54]. Identifying what schools are able and willing to do is essential when launching an initiative (Level 2 actions in Fig. 7). Consistent with literature on implementation of school-based interventions, co-production (i.e., creating and implementing initiatives with schools) is a means to create system changes that has the potential to sustain [51]. Furthermore, our roadmap points out the importance to create a community of practice to enable peer learning and sharing among schools and teachers (Level 3 actions in Fig. 7). These actions can increase the organisational readiness for change [55], which refers to organisational members’ shared resolve to implement a change and shared belief in their collective capability to do so [56]. Our solutions suggest this community of practice can be developed as a learning collaborative to support knowledge exchange among teachers, students, family and wider community partners (e.g., coaches, sports clubs).

Our findings suggest that teachers’ capacity to develop students’ FMS is limited due to a gap in their initial education and ongoing professional development, and this gap must be bridged to improve knowledge and appreciation of FMS. This is in line with the finding from a recent study that surveyed primary school staff in the UK, in which the majority indicated they have low or no perceived knowledge of FMS and do not recall having training on FMS [57]. Our findings highlighted three pillars of quality PE which affects FMS teaching and learning: curriculum, pedagogy, and assessment [44]. These three pillars of quality need to be a priority inclusion in initial teacher education and continuing professional development [58]. One of the solutions suggests initial teacher education should aim to link theory to practice in a way that offers trainees “in-field” experience for enhancement of knowledge and understanding. The solution set also advises the modality of FMS training should be continuous rather than “one off” for in-service teachers, since long-term practice changes are underpinned by ongoing training support [59, 60]. Continuing professional development could be offered as an online option to accommodate teachers’ timetabling challenges; the positive impact of which has been reported in a scale-up of effective FMS intervention [61]. Although the use of online platform needs to be carefully contextualised to meet the need of teachers [62].

According to our findings, the knowledge and efficacy of intervention users and individual delivering programmes can also be enhanced by supporting their capability to adapt the interventions or recommended practice (Level 3 actions in Fig. 7). This implies that all participants are active partners rather than passive receivers of an intervention, and it is by adapting to changing circumstances that learning occurs [63]. This is also supported by research findings from a long-term follow up of a FMS intervention where teacher’s sense of ownership of the programme was encouraged by ongoing adaptations [19]. In this context, Intervention delivery is allowed to and ideally open and adaptive based on a common understanding of principles. This series of options and actions support a sense of both initiative and belonging among participants, which represents two critical mechanisms for uptake and sustained practice (i.e. improving autonomy and relatedness, as described in Self Determination Theory) [64]. This is further reflected in one of the option statements in the current study, where it is noted that teachers need to get the support to tailor interventions so they can also *“learn new skills without feeling left on their own to deliver a project*”.

### Implication for research

A cornerstone of the solution roadmap in the current study is the establishment of a high-quality evidence base, which is needed to frame actions at both policy and practice levels. FMS intervention research to date has generated evidence to help physical educators and teachers plan for successful strategies [3, 65]. Nevertheless, rarely has this been established, embedded, and sustained in the intended settings. By directing attention to the ecological context of FMS intervention research and participation, the roadmap provides researchers with a framework of critical components and players that need to be considered when planning and evaluating an intervention, as well as a list of strategies to improve implementation. There are notable challenges to conducting implementation and sustainability research which include funding and resources constraints, and researcher’s lack of knowledge and incentives [54]. The use of effective planning strategies can ensure resources are well allocated (Level 4 actions in Fig. 7). Notably, resources are not limited to funding – also included are tools, expertise, and skills, as well as sufficient time. Review and empirical evidence in physical activity research suggest that appropriate application of implementation theories/frameworks across the lifespan of an intervention can support programme implementation and sustainability [66–68]. Building upon the roadmap and actions identified in the current study, the CI method can also be used in a local problem situation to identify implementation and sustainability levers to catalyse available resources in efforts to advance local project work. The roadmap also identifies multiple strategies which can be employed to limit the impact of identified barriers, pointing to the importance of implementing solutions at higher levels that are likely to influence solutions at succeeding levels.

The systems of solutions identified across the roadmap highlight that the research process needs to be open, emergent, and reflexive with participants treated as active partners and learners rather than receivers, which includes incorporating participant voices in the formative planning process (Level 3 actions in Fig. 7). Intervention evaluation also needs to consider affective outcomes such as motivation underlying participant engagement in addition to primary intervention outcomes, to understand more complex affective and motivational dynamics as an intervention unfolds. Ultimately, this evidence may contribute to establishing the benchmarks of quality FMS programmes which can be considered in future research and practice.

When planning for intervention translation, researchers also need to consider the economic and societal impacts that may be relevant to stakeholders, as these factors are key for sustainability (Level 4 actions in Fig. 7). The overall impact should be communicated through a variety of channels to spread the word about the benefits of the intervention and new practice. These include preparing intervention champions to demonstrate leadership in the authentic implementation and maintenance of intervention practices [53].

### Strength and limitations

A particular strength of this study is that it is, the first to deploy a meta-analytical CI approach to identify barriers to implementation and sustainability of FMS interventions, and a system of options and an action roadmap to address the complexity of the societal issue. By producing a synthesis from experts across three intervention groups using the CI method, the current study highlights options and an action roadmap that is potentially applicable to a broad variety of FMS intervention contexts where similar implementation and sustainability issues exist.

In response to the COVID-19 pandemic restrictions, this study is one of the first efforts to implement CI online and thus demonstrates the utility of implementing CI via this mode of delivery. Central to CI is the facilitation of systems thinking in a group and management of group dynamics [29]. Adaptation of the CI process to an online format has a few implications. One potential in running CI using a video conferencing tool is the partial restriction on a facilitator’s ability to regulate discussion flow using the full range of verbal and non-verbal cues possible in face-to-face sessions [69]. Specific to Session 3, to prevent technical difficulties, all participants were asked to turn off the camera and to contribute their inputs in turn (e.g., reasoning during ISM structuring) upon the facilitator’s invite. While this turn-taking and facilitator invitation process is similar to face-to-face CI work, and while the verbal reasoning process is central to systems modelling work, in the absence of seeing participants’ non-verbal responses and the associated group dynamic, having the cameras off made it more challenging for the facilitator to ‘step in’ and steer the conversation. In addition, our study sample, although possessing expertise and experience in the domain of FMS interventions (as shown in Table 1), were primarily academics. Future research including a broad range of stakeholders (e.g., head teachers, classroom teachers, parents, and students) is encouraged to further understand barriers to implementation and sustainability of FMS interventions and options to address barriers.

## Conclusions

The current study highlights the complexity of implementation and sustainability of FMS interventions and provides a system of options and a roadmap of actions that help navigate through the complexity. This study contributes to building the knowledge base of strategies required to support research-to-practice translation in FMS interventions. Further application of the CI process and emergent action roadmaps will help researchers, practitioners, and policy makers to design and operationalise future projects in more systemic and relational terms and support more robust implementation and sustainability of FMS interventions at local and national levels.

## Supplementary Information

**Additional file 1.**

## Data Availability

All data generated or analysed during this study are included in this published article and its supplementary information files.

## Abbreviations

FMS: Fundamental Movement Skills
PE: Physical Education
WHO: World Health Organisation

## Footnotes

1 Locations are indicated using letter identifiers rather than naming specific locations.
