# Supplementary material for: Enhancing the implementation and sustainability of fundamental movement skill interventions in the UK and Ireland: lessons from collective intelligence engagement with stakeholders
Source: Int J Behav Nutr Phys Act. 2021 Nov 3;18:144. doi: 10.1186/s12966-021-01214-8 (PMC8565647; doi:10.1186/s12966-021-01214-8)
Supplement: Supplementary file 1 — Additional file 1. [file 12966_2021_1214_MOESM1_ESM.docx]

| 1. Curricular Conflicts | Synthesised option statement |
| --- | --- |
| Theme 1A (Prioritise PE, PA and FMS in schools) | Change school ethos and values around PE through learning workshops and mission documents that promote awareness and understanding of FMS and its impact on core school outcomes including cognitive and social skills |
| Establish and encourage the significance/importance of PE as a core subject and promote the importance of FMS/PA |  |
| Organise workshops or create documents explaining why FMS/PA is important for children and that it actually supports learning on Maths and English |  |
| Change the perception of PE in terms of its identity. It isn't an cogntive based subject (like maths and english) but instead a subject that promotes learning in through and about movement. This shifts it to an ecological subject of learning in development. Through colaborations with researchers, schools will lead to change in policy over an extended period of time. |  |
| Change school ethos around PE and school mission/values |  |
| Promote awareness/understanding/education of FMS and its impacts it can have on core subjects e.g. language, cognition, social skills |  |
| Theme 1B (Assessments and targets in PE) | |
| Demand programme reports from PE and assessment in PE | Establish specific, mandated targets on FMS and PA and demand these to be achieved and reported by schools, in order to direct intervention time and resources and encourage programme uptake by schools |
| Establish specific, mandated targets for all schools to have to achieve; without a specific target, time/resources will always be diverted away from research/intervention |  |
| Boradly speaking if PE had a greater role within the curriculum i.e. had specific targets to be met, schools would be more receptive towards interventions/projects that could help to improve the PA/FMS of children within their school |  |
| Theme 1C (Shared goals) | |
| Identify shared goals between schools and researchers and promote and establish collaborative work of research team with the school and align their aims, research may indirectly support the aims of the school | In advance of programme implementation, generate goals shared, measurable in a collaboration between schools, researchers and policy makers, and build coalitions and partner relationships to support implementation efforts |
| Create common research and school goals/targets and design intervention goals to be measurable by staff and relate their goals |  |
| Conduct research to drive information/knowledge around the measureable goals to achieve a whole of objectives |  |
| Develop sustainable interventions co-designed between teaching staff and researchers so goals are shared, achievable, sustainable and feasible |  |
| Promote collaborations between policy makers and researchers e.g. data on PA/movement competency/weight status |  |
| Develop a mutual understanding of intervention targets before the beginning of the intervention and discuss with schools about possible implications and how to deal with them |  |
| Conduct observations, auditing and evaluating to refine school policies in light of the shared values, ethos and goals |  |
| Theme 1D (Embed movement culture in schools so it doesn’t interfere with other demands) | |
| Promote movement opportunities throughout the whole school day so it is embedded through educating staff/sharing practice of good examples | Use and promote a whole-school approach to embed movement opportunities throughout the whole school day, including curricular, extracurricular, cross-curricular, active transport, and homework |
| Develop a school movement plan/policy to cover curricular and co-curricular, active transport and homework |  |
| Create ways to translate PE content in time that does not interfere with other subject (e.g. making sport material available in recess timein the period when children are learning a specific sport, giving chance to pracctice skills (e.g. juggling) in safe spaces during breaktime between lessons |  |
| Develop cross curricular activities i.e. possible ways to incorporate elements such as literacy/numeracy within PE lessons |  |
| Theme 1E (Impact statement) | |
| Demonstrate a holistic benefit of implementing a movement based curriculum to drive change | Report impact from the programme and disseminate knowledge in relation to quality of life, health, and learning outcomes |
| Disseminate at different levels about the importance of child physical and movement development and potential impact on quality of life and other important heath reated outcomes |  |
| Change school targets based on scientific evidence |  |
| Theme 1F (physical environment) | |
| Audit space use and plan/organise/develop ways to use staff/resources/space more effectively | Evaluate, adapt, and create the physical structures, equipment, and school resources to support programme implementation |
| Encourage explorations of a variety of spaces/environments |  |
| 2. Government and Institutional |  |
| Theme 2A (policy development) | |
| Demand policy development to increase the significance of PE in schools, to the same standard as other core subjects i.e. specific targets to be met and inclusion within OFSTED | Establish a multi-sector task force to develop, implement, and evaluate child health and development policies and programmes that support PE in schools by directing appropriate funding and resources to local councils |
| Set up a task force–multi –education, health to develop, implement and evaluate policy |  |
| Create a policy which prioritise PE as core subject to curriculum by which schools are measured by and promote it |  |
| Develop and implement policy to put child health and development at the forefront by increasing the importance of PE in schools and directing appropriate funding and resources to local councils to build and deliver |  |
| Develop policy and promote, implement and evaluate it |  |
| Theme 2B (build evidence base) | Promote recognition and importance of PE and FMS at national and local level through educating policy holders based on evidence drawn from high quality research |
| Promote understanding at government level, and in turn, school-level understanding of the mental, physical and social benefits of physical activity |  |
| Educate policy holders on the importance by evidence based practice drawn from high quality interventions/evaluations which have all stakeholder input (shared/collective knowledge) |  |
| Encourage recognition of PE and FMS importance by government at national and local level |  |
| Build evidence base for the importance of PE/FMS in children for lifelong health to inform policies |  |
| Theme 2C (promote evidence base) | |
| Promote the evidence/findings from the above task force to change government level policies | Build and communicate robust evidence with stakeholders to encourage uptake of PE and FMS at government level |
| Build evidence for potential support provision of time for PE and FMS |  |
| Encourage all stakeholders to promote the evidence base to policy holders through impact. e.g. working group |  |
| Theme 2D (translate evidence base) | |
| Establish policy frameworks associated with measureable outcomes (evaluation technique) Develop FMS specific targets or outcomes within the curriculum, this would mean that schools would have to make these outcomes a specific target and direct resources to meeting said targets | Translate evidence base into practical solutions coupled with evaluation techniques and measurable outcomes to create clear FMS guidelines, programme methods, and assessments to be embedded in PE curriculum |
| Establish a working group to organise the evidence base to inform evidence building |  |
| Develop evidence based framework and identify areas of future research to build futher base |  |
| Create resources e.g. FMS guidelines/assessments from evidence base which can be embedded into PE curriculum policy |  |
| Create an evidence-based concept which can be brought into and promoted easily as said before (e.g. movement culture) |  |
| Theme 2E (professional development for teachers) | |
| Demand better training for teachers to ensure better structures for intervention sustainability are achieved | Demand and organise better training for teachers |
| Organise appropriate CPD/days to incorporate appropriate teaching training |  |
| Theme 2F (build collaboration between research and policy holders) | |
| Establish more research collaboratoions between government and university bodies to promote joined-up thinking | Build collaborations between research, schools and policy holders to promote joined-up thinking |
| University as a means to reach governmental bodies which listen to their relevant university development |  |
| Build collaborations with schools under a common aim to encourage their uptake |  |
| Theme 2G | |
| Establish appropriate evaluation techniques to identify the efficacy and sustainability of programmes | Improve and change the current evaluation practice to incorporate more appropriate techniques, change the priority of what determines an intervention success and conduct more long term and follow-up evaluation to monitor sustainability |
| Change the priority of what determins an intervention success |  |
| Conduct long, long term evaluation with the same “children+adults” (same participant group) |  |
| Theme 2H (build structures to support sustainability) | |
| Develop structures to ensure sustainability of programmes | Develop structures to support programme sustainability, including developing knowledge hub and partner relationships, educating undergraduates, and promoting programme integration into curriculum |
| Build a “Central Hub” of knowledge and include links for NGB/University etc |  |
| Undergraduate programmes to bring in structure and familiarity with intervention programmes |  |
| Promote integration of the intervention through exisiting curriculum |  |
| 3. Conflicts and Purposes within PE |  |
| Theme 3A (create intervention and its evaluation with stakeholders input) | |
| Conduct stakeholders meetings to clarify intentions of FMS interventions to not create opportunities for interpretation | Conduct stakeholders meetings to clarify intervention aims and results and consult stakeholders on ways to translate intervention findings into practical settings |
| Conduct stakeholders meetings to clarify the meaning of results or provide opportunities for questions/feedback |  |
| Create ideas on the focus points the assess and how to assess (e.g. A delphi poll around PE practitioners and experts) |  |
| To create ways in which those leading the intervention and the PE teachers can discuss and communicate each of the results to better inform the teachers of the findings |  |
| Ask relevant stakeholders whether we need a PE assessment and why - to ascertain the reason behind its creation - assessment, importance of PE in curriculum etc. |  |
| Communication between both parties (researchers and stakeholders) to outline what each will entail and help appreciate the need for both - perhaps when best appropriate to focus on either or |  |
| Theme 3B (Innovation on interventions) | |
| Create resources for promotion of games that develop FMS so that children have fun but also develop FMS | Develop theory-based interventions and resources as well as adapt pedagogical approaches |
| Moving away from direct instruction and towards more novel pedagogical approaches e.g. Nonlinear pedagogy |  |
| Develop an intervention based on achievement goal theory and assess its effect on PMC |  |
| no. 3 - explore existing pedagogies ability to support competence |  |
| Theme 3C (assessment) | |
| Highlighting to children what we are looking at in order to provide context and allow them to reflect on their own progress/performance over the course of an intervention/term | Apply and prioritise PE/skills assessment for children and provide context-specific feedback to allow them to reflect on their progress and performance. |
| Apply testing that is adequate to the population examined and the aims of the testing |  |
| Define what the most common aims are and create a curriculum based on those aims – FMS, fitness, health, games, sportsmanship |  |
| Develop assessments where children’s movement skills are measures and assessed in PE – rather than through reductionist measures that are devoid from context |  |
| Theme 3D (professional development for teachers) | |
| CPD for PE teachers may need to be part of intervention in order to provide further rationale for intervention to teachers | Strengthen CPD for teachers and include intervention and educational aims in the training |
| Providing mode details and material for teachers to clarify the PE aims in the different key stages and how they could be tested (e.g. further material attached to each Key stage educational aim) |  |
| 4. Efficacy and Attitude |  |
| Theme 4A (building structures to support sustainability) | |
| Promote continuity of message of FMS from primary to post-primary years and follow a full life span approach | Create practical and appropriate resources and build structures to promote continuity of FMS messages following a lifespan approach and provide practitioners confidence and rewards to carry out their work |
| Producing documents and sufficient resources and guidance that provide practitioners with confidance to carry out ideas |  |
| Build a rewards system whereby everyone's wellbeing is enhanced when participating in such interventions |  |
| Resources that are sustainable, beneficial, practical and developmentally appropriate through the years |  |
| Theme 4B (professional training) | |
| Greater level of training/CPD for teachers to help them understand the theory and rationale behind what we are trying to do | Strengthen CPD for teachers and include intervention and educational aims in the training |
| Theme 4C (collaborative efficacy) | |
| Co-delivery of projects i.e. led by practitioners and teachers, this way teachers get support with delivery and are able to learn new skills without being left on their own to deliver a project | Provide support for practitioners and teachers to co-lead the delivery of projects |
| 5.Research Challenges | |
| Theme 5A | |
| Establish a cultre of field researchers in undergraduate university programmes | Integrate intervention science and associated field work in undergraduate teaching programmes |
| Theme 5B | |
| Create a teacher feedback method to report fidelity | Establish a feedback method for teachers to report fidelity on programme delivery |
| Theme 5C (collaboration) | |
| Establish links between our department and other departments in a way that helps the resarch via new tools, methods or expertise | Establish cross-disciplinary collaborations in research to access new tools, methods and expertise. |
| Theme | |
| Build relationships with/between different stakeholders in the research process i.e. universities/schools/NGBs. i.e. access to students in return for resources etc. (via reciprocity) | Promote publicity and impact of the intervention programme to potential stakeholders and build reciprocal relationships with them to involve them in future research |
| Build up relationships/networks with local schools to help them understand the work we do and involve them in the research we carry out |  |
| Promote the programme and make it more 'known' to encourage schools and clubs to want to take part |  |
| 6. Intervention Evaluation |  |
| Theme 6A (methodology) |  |
| Create evaluation programmes with minimum 5-year follow-up and sustainability data | Conduct more rigorous and comprehensive evaluation including pilot research, long term follow-up that yields sustainability data, and evaluation of what determines intervention success |
| Conduct pilot studies where possible problems are explored in a small scale |  |
| Change the priority of what determines in intervention success(i.e. currently not enough on "knowledge and understanding" |  |
| Theme 6B |  |
| Encourage integration of programmes and interventions with pre-existing school curriculum and syllables | Encourage integration of programmes and interventions with pre-existing school curriculum and syllables |
| Theme 6C |  |
| Promote the use of common outcome metrics in PA and FMS across all stakehodlers, i.e. joined-up thinking | Promote common outcome metrics in PA and FMS across all stakehodlers |
| Theme 6D |  |
| More inter/intra-university collaborations which could support longer impact and wider joined-up thinking | Promote collaborations between research institutes for wider impact |
| 7. Knowledge and Appreciation |  |
| Theme 7A (create resources) |  |
| Create fun games that children can play, learn and practice at home and in school | Create appropriate resources and disseminate them in different formats to be shared with stakeholders, including guidelines on creating suitable skill learning environments, fun games for children to practice FMS, social marketing of programme benefits on children’s development and skill specific curriculum programmes. |
| Create FMS content using social figures (athletes, influencers etc.) |  |
| Create posters and charts highlighting the effectiveness of interventions/skill acquisition to pu up in schools for students and teachers to see everyday |  |
| Promote messages to encourage FMS/PA links, development and reasons why. |  |
| Create and promote the idea of a "movement culture" to ensure alignment and buy in (via buzzword) |  |
| Create specific curriculum programs for primary teachers to be teaching PE in primary school and creation of courses for teachers to stay aligned with new knowledge in the fie |  |
| Create guidelines School environments/facilities can vary, guidelines for how to create "demanding" environments need to be adaptable |  |
| Create different dissemination documents in different format (e.g. video, written document ) to be shared with school or to be made accessible to stakeholders |  |
| Theme 7B (create learning collaborative) |  |
| Establish teacher/coach/parent/carer etc. prior knowledge of FMS/PA | Create a learning collaborative for stakeholders to share their knowledge and experience regarding FMS and existing FMS resources, as well as to link with researchers to disseminate importance of FMS and best practice |
| Promote a multi-stakeholder approach to an intevention perhaps using health and wellbeing cards, activity journals etc. |  |
| Set up communities of practice to share information etc. |  |
| Create an online forum available to teachers and parents promoting the importance of FMS. Ensure it is shared and publicised in schools and clubs |  |
| Create a central hub of knowledge that is accessbile to all and targeted |  |
| Share existing FMS resources amongst a wider range of stakeholder (clubs, primary, secodary, disabilitiy schools) |  |
| Build a system that to link experts to stakeholders (e.g in some countries politicians regularly meet with researchers) |  |
| Theme 7C (training on FMS and pedagogies) |  |
| Planning effective coaching and teaching programme that nurture appropriate pedagogical practice | Plan and implement effective pre-service and in-service teacher training programme to include relevant pedagogies and techniques, learning workshops on knowledge and understanding of FMS |
| Promote knowledge of relevant peadagoies among teachers when they enter the profession? |  |
| Provide workshop and information transfer to explain the importance of FMS, in order to enhance knowledge and understanding |  |
| Develop CPD and intervention related training - understanding the techniques and how to improvechildren's skills |  |
| Theme 7D (research) |  |
| Develop and implement appropriate research methods to examine enjoyment of FMS interventions | Conduct research on participant understanding of and engagement in intervention programmes and create solutions to overcome perceived barriers and misconceptions |
| Develop conversations on why this is the case? Should their interest be more focused on retaining focus and challenging students - benefit future provision and practice and understanding of children's FMS etc. |  |
| Organise discussions with teaching staff on what can be done to overcome the perceived barriers (discuss why teaching staff have an inadequate appreciation - what are their perceived barriers? What can be done to shift their perceptions?) |  |
| Theme 7E (build structure to support) |  |
| Build the knowledge together at once student, teachers, parents, coaches-all for one type knowledge awareness | Create norms of knowledge building and continuous learning to support students, teachers, parents, and coaches |
| Build a culture where knowledge outweighs "content" |  |
| Establish continuous rather than "one off" specific CPD content for teachers on FMS and PA |  |
| Theme 7F (mobilise parents) |  |
| Role/influence of parents often overlooked in intervention design - what role/information do parents need in relation intervention being delivered? | Expand programme reach to parents and mobilise parental engagement in interventions |
| Create ways to interact with parents without interfering with their time schedule such as online and easy to access platforms where they can access materials and information |  |
| Develop links with parents to promote their engagement in interventions no. 2 - ask parents what would help them engage with the interventions? At what level do they need to engage? Is their engagement purely as gatekeeper to their children? Or should they have a deeper engagement? If the former, what would attract them to engage in the recruitment process? |  |
| Theme 7G (tap into the debate on the correct technique to move) |  |
| The idea of correct technique in children's motor development is misguided. There maybe a more functional ways to navigate the performance environment and children should be nudged toward finding these different solutions | Challenge the idea of correct technique in children’s movement and encourage children to explore under guidance |
| Challenge people's perceptions of what a "wrong technique" is. Is there such a thing as a wrong technique if the outcome goal is achieved? |  |
| 8.Resources and Funding |  |
| Theme 8A (quality PE) | |
| Create a list of what is considered basic necessities for PE? The schools would potentially use some of their PE premium to make sure they have the necessary equipment available. | Create a checklist of essentials for quality PE which guides schools planning on provisions |
| Theme 8B (research planning) | |
| Plan research based on available resources | Conduct research planning based on available resources including proposing suitable research questions, creating cost-effective solutions in research activities such as training teachers to collect research data |
| Plan strategically in the RQ you ask that are balanced against resources |  |
| Plan action-based research where staff within school receives a training anr provides intervention so costs are minimised - potentially training teachers to help researchers in data collection too if feasible |  |
| Create strategies with school to find resousces and time |  |
| Create greater links with research team - provide information and support |  |
| Theme 8C (collaboration) | |
| Create communities of practice where we seek out new knowledge and development together in universities instead of chasing grants. | Create communities of practice among research institutes and consult stakeholders on bids for funding |
| Discussions between research team and stakeholder - potential for bids to be written to obtain further funding - what for? Equipment? Staff? etc. |  |
